# Development and Characterization of UV-Resin Coated Fiber Bragg Gratings

**DOI:** 10.3390/s20113026

**Published:** 2020-05-27

**Authors:** Arnaldo Leal-Junior, Anselmo Frizera, Carlos Marques

**Affiliations:** 1 Graduate Program of Electrical Engineering, Federal University of Espirito Santo, Vitória 29075-910, Brazil; frizera@ieee.org; 2 I3N & Physics Department, Universidade de Aveiro, Campus Universitário de Santiago, 3810-193 Aveiro, Portugal; carlos.marques@ua.pt

**Keywords:** fiber Bragg gratings, UV-curable resins, optical fiber coating, tensile tests, temperature sensor, strain sensor

## Abstract

We report the development and characterizations of a fiber Bragg grating (FBG) sensor coated with different ultraviolet (UV) curable resins. The UV-curable resins were applied on the fiber after the FBG inscription and cured with an UV lamp. One set of samples used the NOA 68 resin and the other used NOA 88. The samples were characterized with respect to the temperature, moisture absorption and strain response. Furthermore, in order to understand the influence of the resin coating on the optical fiber’s mechanical properties, tensile tests were performed with the samples. Results show that all samples presented negligible sensitivity to moisture absorption in the 50-min long tests with the fibers immersed in a container filled with distillated water. Regarding the temperature responses, the coated FBGs presented higher sensitivity (13.84 pm/°C for NOA 88 and 12.76 pm/°C for NOA 68) than the uncoated FBGs due to the thermal expansion of the coatings. In the strain tests, all coated and uncoated samples presented similar sensitivities, but with a larger strain range applied for the coated samples (strains higher than 5500 µε) when compared with the uncoated samples (3500 µε). Moreover, the stress-strain curves of the coated samples indicated a Young’s modulus one order with magnitude lower than the one of the uncoated silica fiber, where the lowest Young’s modulus is 3.84 GPa and was obtained with the NOA 68 coating, which indicates the possibility of obtaining highly sensitive pressure and force sensors.

## 1. Introduction

Optical fiber sensors have been increasingly used in many applications due to advantages such as multiplexing capabilities, intrinsic safe operation, electromagnetic fields immunity, compactness and corrosion resistance [1]. To that extent, it is possible to foresee a widespread use of optical fiber sensing technology, where industrial [2], healthcare [3], chemical [4], structural health monitoring [5] and radiation [6] applications have been proposed. In general, optical fiber sensors are able of measuring physical and chemical parameters using sensing approaches such as intensity variation [7], fiber Bragg gratings (FBGs) [8], interferometers [9] and nonlinear effects [10], among others.

Among the many sensing approaches, FBGs are one of the most common optical fiber sensors due to its inherent high sensitivity and multiplexing capabilities, since it is possible to inscribe dozens of sensors in the same fiber [11]. In addition, FBGs use wavelength-encoded information, i.e., the sensing parameter is obtained from the wavelength shift in the reflected spectrum. This feature leads to a sensor that is insensitive to optical source power fluctuations [12]. The reflection mode operation of the sensors results in simpler sensing assemblies in practical applications. In this case, only one end of the fiber needs to be connectorized to an optical pigtail [13].

Despite the developments in polymer optical fiber (POF) Bragg gratings [14], sensors based on silica fibers still have the highest share on the market and academia. Although the silica fibers have good optical properties such as low optical attenuation and material dispersion [15], mechanical properties of the fiber can limit its application as sensors for mechanical/physical parameters. The silica fiber are brittle and can easily break under transverse strain or axial strains higher than 1.5% [15], which limits the application to small stress and strains. Although the silica fibers have an acrylate coating to increase its mechanical robustness, such coating is generally removed for the FBG inscription. Systems that enable the FBG inscription without removing the coating (such as femtosecond lasers systems) present a high cost when compared with continuous wave or pulsed lasers used in the grating inscription. Moreover, recoating machines enable the coating of optical fibers after the inscription. However, they present high cost and do not enable the in situ recoating of optical fiber.

In order to overcome the material limitations of silica optical fibers, protective coatings are commonly used on the fiber. These coatings include metallic ones to obtain temperature sensors without the influence of mechanical stress [16], where such metallic coatings also enable the use of FBGs in high temperature scenarios [17]. However, if sensor for mechanical measurements (such as force and strain) is concerned, polymer coating are preferable, since they can enhance the operational limits of these sensors [18]. In this case, polymer coating through three-dimensional (3D) printing techniques was proposed in [19]. Furthermore, some polymers have water absorption properties, which can used on the development of FBG-based humidity sensors [20]. It is also worth noting that the integration of FBG sensors in composite structures have been proposed to extend sensor capabilities [21].

Being flexible and easy to use with applications in photonic devices, ultraviolet (UV) curable resins are commonly employed on the connection between polymer optical fibers and silica fibers, for many sensors applications [14]. Recently, the UV resins were used on the development of Fabry-Perot interferometers for sensing applications [22,23]. Despite these recent developments, the UV curable resins are also used as a protection coating for the fiber, where they were also used on the development of FBGs with protective coatings [24]. However, the FBGs in this case presented lower reflectivity, which can harm potential practical applications. For these reasons, a motivation of this work is the development of a technique for FBG coating with low cost and high customizability of both thickness and resin material for the development of FBG sensors with higher sensitivity and/or extended measurement range for thermal and mechanical parameters.

This paper presents the development and characterization of an FBG with protective coatings made of UV curing resins to overcome the issues of FBGs in silica fiber for mechanical sensing. The proposed method is simple, low cost, providing robust coating of the fiber. In addition, there is the possibility of in-situ application of such coatings, which is not possible with the majority of the coating approaches for optical fibers. As another contribution of this work, the FBG with protective coating is characterized for different sensing applications, where the temperature and strain responses as well as the moisture absorption are analyzed for different UV curable resins as protective coatings. Moreover, the stress-strain curves of the fibers with different protective coatings are also obtained and analyzed.

## 2. Experimental Setup

**Figure 1:**
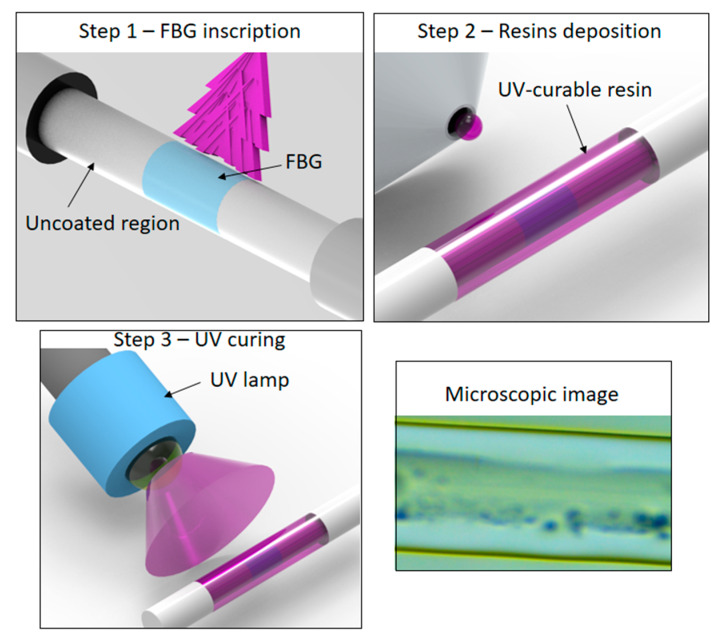
Schematic representation and microscopic image of the UV-curable resins coating on the fiber Bragg grating (FBG).

The FBGs used in this work were inscribed in a photosensitive single mode fiber GF1B (Thorlabs, USA) using a nanosecond pulsed Nd:YAG laser (LOTIS TII LS-2137U Laser) with 8 ns pulse time via the phase mask technique [25]. For the grating’s inscription, the acrylate protection of the silica fiber is striped, which leads to increase on the fiber brittleness, since there is only the glass material without the polymeric protection. Then, we applied the UV curable resin in the FBG region (without the acrylate protection). Two resins were used, NOA 88 and NOA 68 (Norland, Lincoln, NE, USA), in order to verify the influence of the resin’s properties in the sensor responses. These resins were chosen due to their different viscosities (5000 cps and 250 cps for NOA 68 and NOA 88, respectively) in order to analyze the sensors responses at different coating conditions. The resin is radially applied on the fiber and the UV curing occurs with a UV lamp CS2010 (Thorlabs, Newton, NJ, USA) with irradiance of 27 W/cm^2^ at 365 nm. The process of applying the resin on the fiber comprises of filling a container with the resin and dip the fiber in the container for a few seconds. Then, UV curing is performed. The curing is performed in about 1 min in order to guarantee a complete polymerization of the resin and leads to a coated fiber with about 600-µm diameter obtained from the dipping and curing times. The coating thickness and uniformity are guaranteed by the control of the variables in the dip coating of the UV curing resin. In this case, the dipping time is controlled as well as the dipping length and withdraw speed, which remain constant for all fabricated samples. Moreover, the uniformity of the coating is obtained by rotating the sample without significant variations in the angular velocity and distance between the sample and the UV lamp. Figure 1 shows a schematic representation that summarizes the described process of applying the UV curable resin coating on the optical fiber. Moreover, a microscopic image of the coated fiber is shown in Figure 1.

The temperature response of the FBGs with UV curable resins is analyzed by means of positioning all the produced samples inside a climatic chamber 1/400 ND (Ethik Technology, Brazil) and a temperature variation ranging from 30 °C to 55 °C in 5 °C steps was applied at all samples. For comparison purposes, an FBG in silica fiber without coating (acrylate protection) is tested. From the temperature variation tests, it is possible to compare the temperature sensitivity and response time of each sample. The goal is to evaluate if the use of a protective coating with an adhesive resin can lead to different temperature sensitivity in the FBG (when compared with the fiber without coating) due to thermal expansion of the resin [26]. In addition, the use of a UV resin as coating in the optical fiber leads to an increase on the fiber diameter at the region with the coating (when compared with the uncoated fiber); such an increase can lead to longer response times for temperature variation, which will be dependent not only on the coating thickness, but also on the thermal conduction capabilities of the material used as coating [27].

As many polymers have moisture absorption, we investigated the wavelength shift of the FBGs with the different coatings when the fiber is immersed in a container filled with distillated water. The fibers were positioned in the container and the wavelength shift was monitored for about 1 h. The optical interrogator sm125 (Micron Optics, Atlanta, GA, USA) was used on the reflected spectra acquisition and wavelength shift monitoring with an acquisition frequency of 2 Hz and a wavelength resolution of 1 pm.

In order to verify if there is any variation of the mechanical features of the fiber with different UV curable resins coatings, tensile tests were performed in the fibers with coatings using a universal testing machine (Biopdi, São Carlos, Brazil). All samples were tested and from the stress-strain curves, the material Young’s modulus and yield stress were evaluated for each case. In this case, the yield stress is the stress in which the material begins its plastic deformation, after the linear deformation. In the stress-strain curve, it is the region where the material does not show a linear relation between stress and strain. The Young’s modulus is estimated from the slope of the stress-strain curve in the linear region.

**Figure 2:**
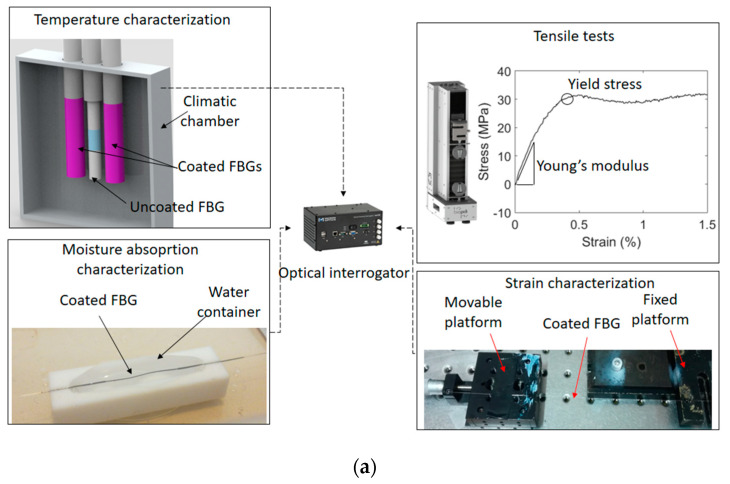

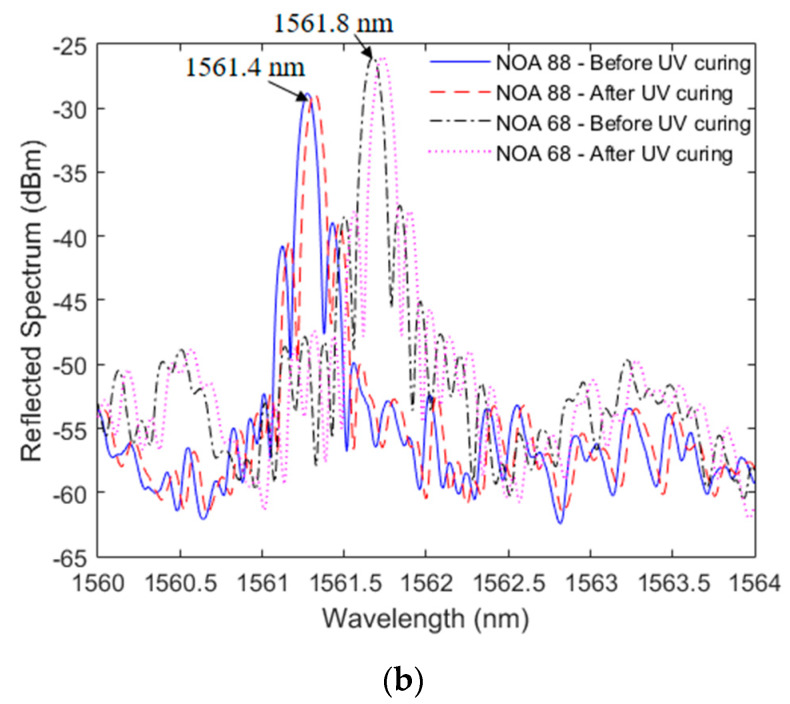
(**a**) Schematic representation of the characterization tests performed in the coated and uncoated FBGs. (**b**) Reflected spectra of the FBG samples before and after the UV curing.

Finally, the strain responses of the FBG sensors with and without coatings are analyzed in a strain range from 0 to 5500 µε. The test is performed by means of gluing the fiber using a cyanoacrylate glue with one end in a fixed and the other in a movable platform. Thus, axial strain is applied on the fibers and we analyze the sensitivity and the strain range in the fibers. It is expected that the fiber with the UV resins coatings will present higher strain limits when compared with the uncoated sample. In order to summarize the tests performed in the FBGs, Figure 2a shows the schematic representation of each performed test, which include strain, temperature and moisture absorption as well as the tensile tests for the fiber mechanical properties. In Figure 2b, the reflected spectra of the samples before and after the UV curing of NOA 68 and 88 resins are shown, where it can be inferred that the UV resin coating does not lead to significant changes in the reflected spectra. In the analyzed cases, the resin coating only resulted in a small wavelength shift of 0.06 nm and 0.05 nm for NOA 68 and 88, respectively.

## 3. Results and Discussion

**Figure 3:**
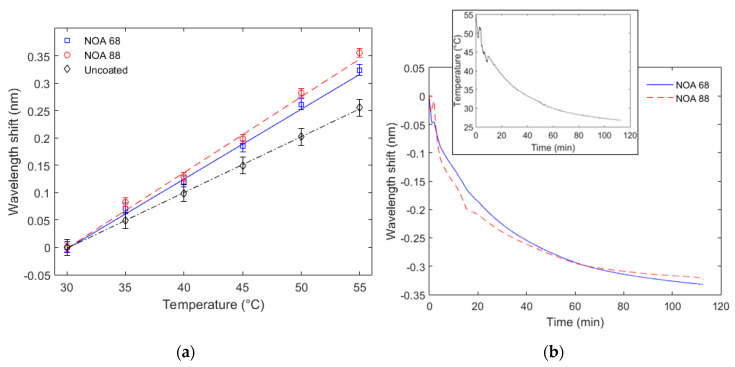
Temperature responses of each coated and uncoated sample. (**a**) Linear regression of wavelength shifts as a function of the temperature. (**b**) Wavelength shift as a function of time for the temperature decrease in both samples. Figure inset shows the temperature variation as a function of the time.

Figure 3 shows the results obtained in the temperature tests for each sample, i.e., uncoated, NOA 88 and NOA 68 coated FBGs. In Figure 3a, the linear regression of the wavelength shift as a function of the temperature is presented with the standard deviation of three consecutive tests, whereas the temperature decrease is presented in Figure 3b, where the sensor response time can be inferred from the slope of the temperature decrease curve.

The results in Figure 3a indicate a higher temperature sensitivity for the sample with NOA 88 coating (13.84 pm/°C), which is close to the one obtained for NOA 68 coating (12.76 pm/°C). Both sensitivities are higher than the one for the uncoated FBG (10.19 pm/°C), which indicates that the thermal expansion of the coatings under temperature variation plays an important role on the coated sensors temperature sensitivity. Although the manufacturer did not show the thermal expansion coefficient of each resin in the datasheet, it is possible to infer that NOA 88 has a higher thermal expansion coefficient due to the higher temperature sensor sensitivity when such resin is used as optical fiber coating. However, when the sensors’ linearities are compared, the uncoated FBG presented a determination coefficient (R^2^) of 0.9999, which is higher than the ones obtained on the coated FBGs, 0.9987 and 0.9973 for NOA 68 and NOA 88, respectively. The lower linearity of the coated samples is related to some nonlinearities in the thermal expansion of the resins as well as some non-uniformities in the interface between the resin and optical fiber that can lead to non-uniform strain on the optical fiber when the resin experiences the thermal expansion. Nevertheless, all samples presented a R^2^ higher than 0.99, which indicates a linear response and leads to low errors in temperature estimation.

Regarding to the temperature decreasing tests, the climatic chamber is turned off when its temperature is at about 55 °C and the temperature decreased to the ambient temperature (about 28 °C) for almost 115 min as shown in Figure 3b. In order to provide a comparison between the temperature decrease and the wavelength shift as a function of the time, Figure 3b inset shows the temperature variation during the cooling test, where it is possible to observe that the sensors have the same pattern as the temperature decreases, indicating similar linearity as found in Figure 3a. The wavelength shift of the FBGs coated with both resins is monitored during the cooling in the climatic chamber, the results can be related to the response time of each sensor. The NOA 88 sample presented a higher wavelength shift as a function of time in the first 20 min, which indicated a faster temperature response in this period, whereas the NOA 68 sample presented a higher wavelength shift after 60 min. Besides the coating thickness, which was similar for both samples (about 600 µm), the differences, in terms of the temperature response time, are also due to the thermal conduction coefficient of each resin, where the resin with the highest thermal conduction coefficient results in the lowest response time. Comparing Figure 3a,b, it is possible to observe that NOA 68 has a 0.32 nm in both conditions (increasing and decreasing temperature), which indicates the reversibility of the sensor, whereas NOA 88 sample presented an error of 0.02 nm. Such error may be related to the resin thickness and distribution along the FBG region. In this case, the NOA 68 sample is the preferable one when a temperature response for dynamic application is needed (or that requires a faster temperature response and reversibility).

**Figure 4:**
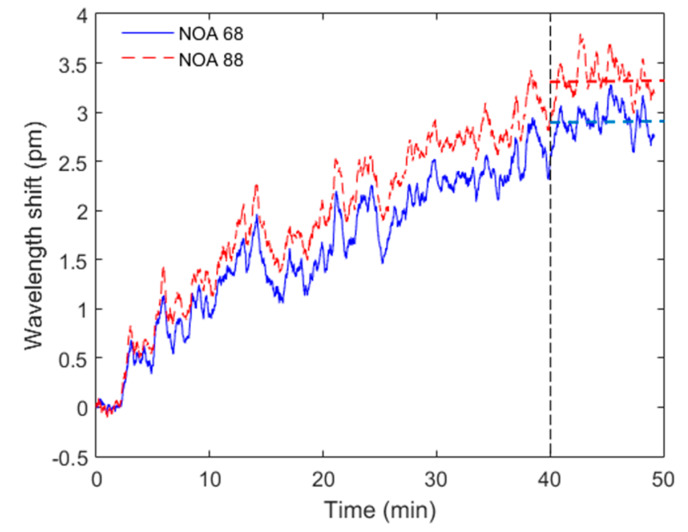
Moisture absorption tests for the NOA 68 and NOA 88 coated FBGs immersed in a fluid container for 50 min.

After the temperature characterization, moisture absorption tests are performed in the coated samples. The results for a 50-min water immersion are presented in Figure 4, where the test was performed at constant temperature of 28 °C and both fibers were immersed in the same fluid at the same time.

The results of Figure 4 indicate a low water absorption of both samples, where the maximum variation was below 4 pm. The NOA 88 sample presented a slightly higher water absorption than the NOA 68 coating. However, considering the low values of both samples, one can consider the water absorption negligible in both cases. Despite this low water absorption, both samples presented a stabilization time of about 40 min; after that, the wavelength shift remains almost constant at each analyzed case, as indicated in Figure 4.

**Figure 5:**
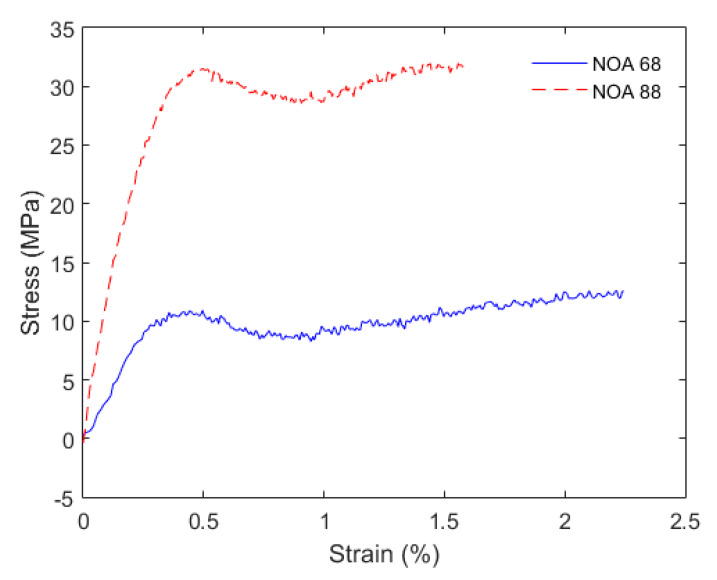
Stress-strain curves of the tensile tests performed with the coated samples.

For mechanical sensors analysis, the tensile tests provide important information regarding the operation limits as well as the tensile modulus of each sample, where a lower Young’s modulus indicates the possibility of producing force or stress sensors with higher sensitivity. The stress-strain curves for each coating condition are presented in Figure 5, where the tests were made with the same strain rate (10 mm/min) for all samples.

The stress-strain curves indicated a yield stress of about 11 MPa for the FBG with NOA 68 coating and 32 MPa for the one coated with NOA 88. Such differences are related to the mechanical properties of each resin (after full cure of the resin). In addition, a Young’s modulus of 3.82 GPa and 7.07 GPa were found for the NOA 68 and NOA 88 samples, respectively. These Young’s moduli are one order of magnitude lower than the ones obtained with uncoated silica fiber (about 70 GPa [28]). Such difference in the moduli are also due to the differences in the mechanical properties of the resins. The NOA 68 has a Young’s modulus of about 137.9 MPa [29], whereas the NOA 88 has a higher Young’s modulus of 903.2 MPa [30]. Thus, the combination between the silica fiber and the resins leads to the reduction of the Young’s modulus, which is proportional not only to the modulus of each material, but also to the thickness of each layer. For this reason, the layer thickness of each resin also influences on the Young’s modulus variation when compared with the uncoated fiber. It is worth noting that the Young’s modulus of the coated fiber is close to the one of POFs, especially for the NOA 68 coating [14], which indicates the possibility of obtaining force and pressure sensors with high sensitivities. As is also shown in Figure 5, the NOA 68 coating can withstand higher strains, which also leads to a sensor with higher dynamic range for strain sensing.

**Figure 6:**
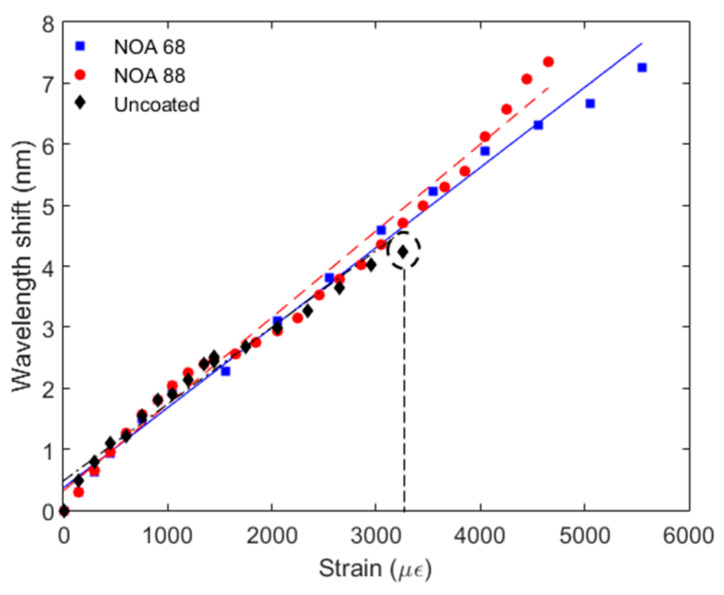
Strain responses of the uncoated, NOA 68 and NOA 88 coated samples.

In order to verify the behavior of each coated FBG as a function of the strain, axial elongations were applied to the fibers in a large range, where the tests were divided in a first step in which lower strains are applied (about 50 µε) and in a second step, where larger strains are applied on the fibers until their breakage. For comparison purposes, the same tests are employed in an uncoated FBG, and the results are presented in Figure 6.

Regarding Figure 6, all samples presented a similar strain sensitivity with mean value and standard deviation of 1.30 ± 0.08 pm/µε. The NOA 88 sample presented a slightly higher strain sensitivity than the other samples, where a sensitivity of 1.40 pm/µε was obtained. In addition, each sample presented similar linearity, where the mean and standard deviation of the R2 is 0.991 ± 0.004, which can be regarded as a high linearity for all sensors. This variation in linearity can be attributed to the high range used in the experiments, where some minor deviations on the sensors positioning and non-uniformities on the cyanoacrylate glue insertion in the interface between the fiber and the translation stage, can result in reduction of sensors linearities as well as in minor differences between the linearity of each sample. Despite the similar linearity and sensitivity, the samples presented different operation ranges, where the uncoated sample broke at an axial strain of about 3500 µε, whereas the other samples continued to operate until a strain of about 5500 µε. It is worth noting that the NOA 68 and NOA 88 were still able of operating in strains higher than 5500 µε. However, the wavelength shift was too high and they were outside the optical interrogator wavelength range. For this reason, we limit the test to 5500 µε. Nevertheless, the goal of this experiment was to verify the assumption that the coated FBG presents higher strain limits (and measurement range) than the uncoated FBG. Thus, even with such limited axial strain it was able to verify that the coated FBG can withstand an axial strain almost two times higher than the one of the uncoated FBG.

The comparison between the uncoated and coated samples show a superior performance in terms of sensitivity and measurement range for all analyzed parameters. In the temperature tests, both coated samples presented a high sensitivity than the uncoated FBG. Furthermore, a measurement range almost two times higher than the one of the uncoated sample was found in the axial strain tests, indicating their feasibility in large strain measurement applications such as in movement analysis, robotics and in some structural health monitoring applications. It is also worth to mention that the tensile tests show a Young’s modulus reduction of at least 10 times when compared with the uncoated samples, which indicate the possibility of achieving higher sensitivities in mechanical parameters measurement such as force, stress and pressure.

## 4. Conclusions

This paper presented the development and characterization of FBGs coated with UV-curable resins. The FBGs were inscribed using the phase mask technique and the UV resins were applied in the uncoated region of the fiber, in which the FBG was inscribed: one sample with the NOA 68 and the other with NOA 88. Then, the resins were cured using a UV lamp. The samples were characterized with respect to the temperature response, moisture absorption and strain sensitivity and range. In addition, tensile tests were performed on the samples and the stress-strain curves were compared. The results show higher temperature sensitivity of the coated samples when compared with the uncoated one due to the thermal expansion coefficient of the resins, where the NOA 88 sample showed the highest temperature sensitivity (13.84 pm/°C). The moisture absorption tests indicated a negligible sensitivity for both samples, where the wavelength shift of the samples immersed in a container filled with water was as low as 4 pm. In addition, the stress-strain tests indicated a lower Young’s modulus of the NOA 68 sample (3.82 GPa—Comparable with the Young’s modulus of POF technology). However, the NOA 88 sample also showed a Young’s modulus (7.07 GPa) one order of magnitude lower than the one of the uncoated silica fiber (70 GPa). Therefore, the FBGs coated with UV curable resins are an interesting alternative for high sensitive pressure and force sensing. For the strain sensing, all coated and uncoated samples showed similar sensitivity and linearity, but with a larger strain range for the coated fibers, which were able to operate in strains as high as 5500 µε when compared with the uncoated sample. Thus, the results presented in this work indicate the feasibility of using UV curing resins as a low-cost, highly customizable and simple method for FBG coating, especially when compared with recoating machines, which provides advantages over the uncoated fibers in many sensing applications. Following this positive feedback, future works include the investigation of more resins as well as unveil the tradeoff between the coating layer thickness, material and sensor performance.
